# Poly[[bis­(μ-4,4′-bipyridyl-κ^2^ *N*:*N*′)bis­(thio­cyanato-κ*N*)manganese(II)] diethyl ether disolvate]

**DOI:** 10.1107/S1600536810021665

**Published:** 2010-06-16

**Authors:** Mario Wriedt, Inke Jess, Christian Näther

**Affiliations:** a Institut für Anorganische Chemie, Christian-Albrechts-Universität Kiel, Max-Eyth-Strasse 2, 24098 Kiel, Germany

## Abstract

In the title compound, {[Mn(NCS)_2_(C_10_H_8_N_2_)_2_]·2C_4_H_10_O}_*n*_, the Mn^II^ ion is coordinated by four *N*-bonded 4,4′-bipyridine (bipy) ligands and two *N*-bonded thio­cyanate anions in a distorted octa­hedral coordination geometry. The asymmetric unit consists of one Mn^II^ ion and two bipy ligands each located on a twofold rotation axis, as well as one thio­cyanate anion and one diethyl ether mol­ecule in general positions. In the crystal structure, the metal centers with terminally bonded thicyanate anions are bridged by the bipy ligands into layers parallel to (001). The diethyl ether solvent mol­ecules occupy the voids of the structure.

## Related literature

For general background to thermal decomposition reactions as an alternative tool for the discovery and preparation of new ligand-deficient coordination polymers with defined magnetic properties, see: Wriedt & Näther (2009*a* ▶,*b* ▶); Wriedt *et al.* (2009*a* ▶,*b* ▶). For the isotypic cobalt(II) structure, see: Lu *et al.* (1997 ▶).
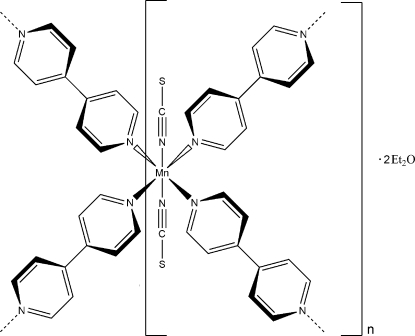


## Experimental

### 

#### Crystal data

[Mn(NCS)_2_(C_10_H_8_N_2_)_2_]·2C_4_H_10_O
*M* *_r_* = 631.71
Monoclinic, 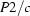

*a* = 11.702 (2) Å
*b* = 11.6391 (18) Å
*c* = 13.424 (2) Å
β = 106.75 (2)°
*V* = 1750.8 (5) Å^3^
*Z* = 2
Mo *K*α radiation
μ = 0.53 mm^−1^
*T* = 230 K
0.22 × 0.14 × 0.07 mm

#### Data collection

Stoe IPDS-1 diffractometer
Absorption correction: numerical (*X-SHAPE* and *X-RED32*; Stoe & Cie, 2002 ▶) *T*_min_ = 0.912, *T*_max_ = 0.968
11086 measured reflections
2954 independent reflections
2446 reflections with *I* > 2σ(*I*)
*R*_int_ = 0.134

#### Refinement

*R*[*F* ^2^ > 2σ(*F* ^2^)] = 0.073
*wR*(*F* ^2^) = 0.206
*S* = 1.07
2954 reflections
191 parameters
H-atom parameters constrained
Δρ_max_ = 0.72 e Å^−3^
Δρ_min_ = −1.37 e Å^−3^

### 

Data collection: *X-AREA* (Stoe & Cie, 2002 ▶); cell refinement: *X-AREA*; data reduction: *X-AREA*; program(s) used to solve structure: *SHELXS97* (Sheldrick, 2008 ▶); program(s) used to refine structure: *SHELXL97* (Sheldrick, 2008 ▶); molecular graphics: *SHELXTL* (Sheldrick, 2008 ▶); software used to prepare material for publication: *SHELXTL*.

## Supplementary Material

Crystal structure: contains datablocks I, global. DOI: 10.1107/S1600536810021665/hy2314sup1.cif

Structure factors: contains datablocks I. DOI: 10.1107/S1600536810021665/hy2314Isup2.hkl

Additional supplementary materials:  crystallographic information; 3D view; checkCIF report

## Figures and Tables

**Table 1:** Selected bond lengths (Å)

Mn1—N21	2.181 (4)
Mn1—N12^i^	2.277 (4)
Mn1—N11	2.300 (4)
Mn1—N1	2.312 (3)

Symmetry code: (i) 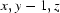.

**Fig. 1.**
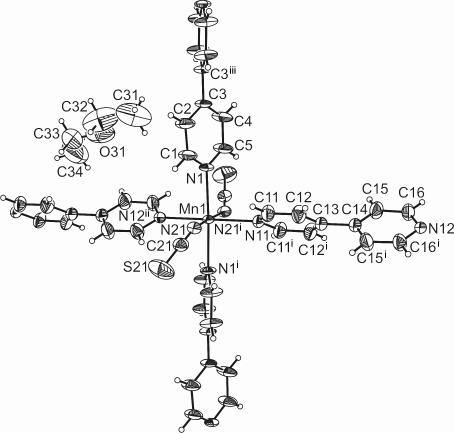
Structure of the title compound with displacement ellipsoids drawn at the 50% probability level. [Symmetry codes: (i) -x+1, y, -z+3/2; (ii) x, y-1, z; (iii) -x, y, -z+3/2.]

**Fig. 2.**
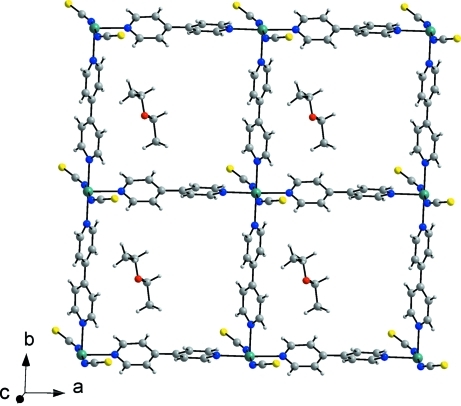
A single layer in the title compound with view approximately along the crystallographic c axis.

## supplementary crystallographic information

### Comment

Recently, we are interested in thermal decomposition reactions as an alternative tool for the discovering and preparation of new ligand-deficient coordination polymers with defined magnetic properties (Wriedt & Näther, 2009a,b; Wriedt *et al.*, 2009a,b). In our ongoing investigation on the synthesis, structures and properties of such compounds we have reacted manganese(II) chloride, potassium thiocyanate and 4,4-bipyridine (bipy). In this reaction single crystals of the title compound were grown.

The title compound (Fig. 1) represents a two-dimensional layered coordination polymer, in which the Mn^II^ atom is coordinated by four bipy ligands and two thiocyanate anions in an octahedral coordination mode. The crystal structure is isotypic to its cobalt(II) analogue (Lu *et al.*, 1997). In the crystal structure the metal atoms are bridged by the bipy ligands into layers with terminally N-bonded thicyanate anions. The layers are stacked perpendicular to the crystallographic *c* axis in order that the metal atoms in one layer sit above or below the squares formed by the metal atoms of the adjacent layers. By this arrangement voids are formed in which the diethyl ether molecules are located (Fig. 2).  The MnN_6_ octahedron is markedly distorted with four long Mn—N_bipy_ distances in the range of 2.277 (4) to 2.312 (4) Å and two short Mn—NCS distances of 2.181 (4) Å (Table 1). The angles arround the metal atoms range between 88.27 (8) to 91.73 (8) and 176.54 (16) to 180°. The pyridyl rings of the bipy ligands form dihedral angles of 51.2 (1) and 52.6 (1)°, respectively. The shortest intra- and interlayer Mn···Mn distances amount to 11.6391 (6) and 8.3198 (11) Å, respectively.

### Experimental

MnCl_2_ (117.0 mg, 0.93 mmol) and KNCS (180.8 mg, 1.86 mmol) obtained from Alfa Aesar were dissolved in a mixture of 10 ml water and 15 ml ethanol. This mixture was layered with a solution of 4,4-bipyridine (306.3 mg, 2 mmol) in 10 ml diethyl ether. After one day colourless block-shaped single crystals of the title compound were grown at the phase interface.

### Refinement

The H atoms were located in a difference Fourier map but were positioned with idealized geometry and refined using a riding model, with C—H = 0.94 (aromatic), 0.98 (methylene) and 0.97 (methyl) Å and with *U*_iso_(H) = 1.2(1.5 for methyl)*U*_eq_(C).

### Crystal data

**Table:**

[Mn(NCS)_2_(C_10_H_8_N_2_)_2_]·2C_4_H_10_O	*F*(000) = 662
*M**_r_* = 631.71	*D*_x_ = 1.198 Mg m^−^^3^
Monoclinic, *P*2/*c*	Mo *K*α radiation, λ = 0.71073 Å
Hall symbol: -P 2yc	Cell parameters from 11086 reflections
*a* = 11.702 (2) Å	θ = 2.4–25.0°
*b* = 11.6391 (18) Å	µ = 0.53 mm^−^^1^
*c* = 13.424 (2) Å	*T* = 230 K
β = 106.75 (2)°	Block, colourless
*V* = 1750.8 (5) Å^3^	0.22 × 0.14 × 0.07 mm
*Z* = 2	

### Data collection

**Table:**

Stoe IPDS-1 diffractometer	2954 independent reflections
Radiation source: fine-focus sealed tube	2446 reflections with *I* > 2σ(*I*)
graphite	*R*_int_ = 0.134
ω scans	θ_max_ = 25.0°, θ_min_ = 2.4°
Absorption correction: numerical (*X-SHAPE* and *X-RED32*; Stoe & Cie, 2002)	*h* = −13→13
*T*_min_ = 0.912, *T*_max_ = 0.968	*k* = −13→13
11086 measured reflections	*l* = −15→15

### Refinement

**Table:**

Refinement on *F*^2^	Secondary atom site location: difference Fourier map
Least-squares matrix: full	Hydrogen site location: inferred from neighbouring sites
*R*[*F*^2^ > 2σ(*F*^2^)] = 0.073	H-atom parameters constrained
*wR*(*F*^2^) = 0.206	*w* = 1/[σ^2^(*F*_o_^2^) + (0.1115*P*)^2^ + 1.3719*P*] where *P* = (*F*_o_^2^ + 2*F*_c_^2^)/3
*S* = 1.07	(Δ/σ)_max_ < 0.001
2954 reflections	Δρ_max_ = 0.72 e Å^−^^3^
191 parameters	Δρ_min_ = −1.37 e Å^−^^3^
0 restraints	Extinction correction: *SHELXL97* (Sheldrick, 2008), Fc^*^=kFc[1+0.001xFc^2^λ^3^/sin(2θ)]^-1/4^
Primary atom site location: structure-invariant direct methods	Extinction coefficient: 0.034 (7)

### Fractional atomic coordinates and isotropic or equivalent isotropic displacement parameters (Å^2^)

**Table:**

	*x*	*y*	*z*	*U*_iso_*/*U*_eq_	
Mn1	0.5000	0.71119 (6)	0.7500	0.0284 (3)	
N1	0.3018 (2)	0.7070 (3)	0.7479 (3)	0.0385 (8)	
C1	0.2710 (3)	0.6632 (5)	0.8264 (4)	0.0557 (13)	
H1	0.3317	0.6340	0.8829	0.067*	
C2	0.1544 (4)	0.6575 (5)	0.8310 (4)	0.0617 (15)	
H2	0.1373	0.6249	0.8892	0.074*	
C3	0.0633 (3)	0.7003 (4)	0.7491 (4)	0.0408 (10)	
C4	0.0940 (3)	0.7447 (5)	0.6646 (4)	0.0577 (13)	
H4	0.0352	0.7725	0.6061	0.069*	
C5	0.2141 (3)	0.7470 (5)	0.6684 (4)	0.0559 (13)	
H5	0.2346	0.7788	0.6115	0.067*	
N11	0.5000	0.9088 (4)	0.7500	0.0367 (11)	
N12	0.5000	1.5156 (3)	0.7500	0.0354 (11)	
C11	0.4781 (4)	0.9689 (3)	0.8272 (4)	0.0448 (10)	
H11	0.4624	0.9285	0.8824	0.054*	
C12	0.4775 (4)	1.0865 (4)	0.8300 (4)	0.0498 (11)	
H12	0.4619	1.1249	0.8862	0.060*	
C13	0.5000	1.1484 (4)	0.7500	0.0384 (13)	
C16	0.4344 (4)	1.4556 (4)	0.6684 (4)	0.0488 (11)	
H16	0.3880	1.4963	0.6104	0.059*	
C15	0.4314 (4)	1.3368 (4)	0.6652 (4)	0.0509 (11)	
H17	0.3837	1.2982	0.6063	0.061*	
C14	0.5000	1.2758 (4)	0.7500	0.0390 (13)	
N21	0.5534 (3)	0.7169 (3)	0.9196 (3)	0.0386 (8)	
C21	0.6253 (3)	0.6927 (3)	0.9963 (4)	0.0412 (10)	
S21	0.72754 (14)	0.65840 (19)	1.10210 (13)	0.0900 (6)	
C31	0.0822 (10)	0.3656 (13)	0.6206 (10)	0.170 (6)	
H31A	0.1479	0.4018	0.6020	0.255*	
H31B	0.0756	0.3981	0.6853	0.255*	
H31C	0.0086	0.3787	0.5659	0.255*	
C32	0.1059 (12)	0.2324 (15)	0.6347 (9)	0.172 (6)	
H32A	0.1679	0.2177	0.7002	0.206*	
H32B	0.0329	0.1930	0.6375	0.206*	
O31	0.1431 (5)	0.1896 (8)	0.5502 (5)	0.138 (3)	
C33	0.1686 (9)	0.0670 (10)	0.5534 (11)	0.146 (5)	
H33A	0.0974	0.0238	0.5554	0.175*	
H33B	0.2328	0.0489	0.6166	0.175*	
C34	0.2049 (11)	0.0329 (12)	0.4606 (12)	0.167 (5)	
H34A	0.1368	0.0383	0.3991	0.251*	
H34B	0.2341	−0.0456	0.4688	0.251*	
H34C	0.2676	0.0837	0.4530	0.251*	

### Atomic displacement parameters (Å^2^)

**Table:**

	*U*^11^	*U*^22^	*U*^33^	*U*^12^	*U*^13^	*U*^23^
Mn1	0.0147 (4)	0.0265 (5)	0.0448 (6)	0.000	0.0100 (3)	0.000
N1	0.0135 (13)	0.0482 (19)	0.057 (2)	0.0019 (12)	0.0146 (14)	0.0072 (15)
C1	0.0196 (17)	0.087 (3)	0.060 (3)	0.0062 (19)	0.0109 (18)	0.025 (3)
C2	0.0233 (18)	0.101 (4)	0.064 (3)	0.005 (2)	0.0176 (19)	0.032 (3)
C3	0.0130 (17)	0.060 (2)	0.051 (3)	0.0018 (15)	0.0119 (15)	0.0015 (19)
C4	0.0209 (17)	0.103 (4)	0.050 (3)	0.012 (2)	0.0110 (17)	0.011 (3)
C5	0.0220 (18)	0.095 (4)	0.054 (3)	0.011 (2)	0.0174 (18)	0.019 (3)
N11	0.034 (2)	0.026 (2)	0.054 (3)	0.000	0.018 (2)	0.000
N12	0.0246 (19)	0.025 (2)	0.055 (3)	0.000	0.0101 (19)	0.000
C11	0.057 (2)	0.032 (2)	0.055 (3)	0.0032 (18)	0.030 (2)	0.0031 (18)
C12	0.065 (3)	0.036 (2)	0.058 (3)	0.004 (2)	0.032 (2)	−0.0027 (19)
C13	0.034 (2)	0.028 (3)	0.054 (4)	0.000	0.013 (2)	0.000
C16	0.050 (2)	0.032 (2)	0.055 (3)	−0.0014 (18)	0.0004 (19)	0.0048 (18)
C15	0.056 (2)	0.034 (2)	0.054 (3)	−0.0069 (19)	0.002 (2)	−0.0031 (19)
C14	0.036 (3)	0.028 (3)	0.056 (4)	0.000	0.018 (3)	0.000
N21	0.0276 (15)	0.0389 (18)	0.049 (2)	0.0025 (12)	0.0111 (15)	0.0026 (14)
C21	0.034 (2)	0.044 (2)	0.049 (3)	0.0014 (16)	0.0167 (19)	−0.0025 (18)
S21	0.0624 (9)	0.1362 (16)	0.0578 (12)	0.0265 (9)	−0.0047 (7)	0.0143 (9)
C31	0.111 (8)	0.246 (16)	0.144 (11)	0.028 (9)	0.022 (7)	−0.056 (11)
C32	0.136 (9)	0.308 (19)	0.079 (8)	−0.067 (11)	0.041 (7)	−0.028 (10)
O31	0.078 (3)	0.235 (9)	0.093 (5)	−0.005 (4)	0.010 (3)	0.028 (5)
C33	0.089 (6)	0.131 (8)	0.204 (14)	0.015 (6)	0.020 (7)	0.061 (8)
C34	0.134 (9)	0.179 (11)	0.198 (13)	0.057 (8)	0.062 (9)	0.020 (10)

### Geometric parameters (Å, °)

**Table:**

Mn1—N21	2.181 (4)	C13—C12^iii^	1.380 (5)
Mn1—N12^i^	2.277 (4)	C13—C14	1.483 (7)
Mn1—N11	2.300 (4)	C16—C15	1.383 (6)
Mn1—N1	2.312 (3)	C16—H16	0.9400
N1—C1	1.311 (6)	C15—C14	1.385 (5)
N1—C5	1.333 (6)	C15—H17	0.9400
C1—C2	1.385 (5)	C14—C15^iii^	1.385 (5)
C1—H1	0.9400	N21—C21	1.161 (6)
C2—C3	1.386 (6)	C21—S21	1.621 (5)
C2—H2	0.9400	C31—C32	1.577 (18)
C3—C4	1.384 (7)	C31—H31A	0.9700
C3—C3^ii^	1.488 (6)	C31—H31B	0.9700
C4—C5	1.392 (5)	C31—H31C	0.9700
C4—H4	0.9400	C32—O31	1.418 (13)
C5—H5	0.9400	C32—H32A	0.9800
N11—C11	1.334 (5)	C32—H32B	0.9800
N11—C11^iii^	1.334 (5)	O31—C33	1.455 (12)
N12—C16	1.338 (5)	C33—C34	1.482 (16)
N12—C16^iii^	1.338 (5)	C33—H33A	0.9800
N12—Mn1^iv^	2.277 (4)	C33—H33B	0.9800
C11—C12	1.370 (6)	C34—H34A	0.9700
C11—H11	0.9400	C34—H34B	0.9700
C12—C13	1.380 (5)	C34—H34C	0.9700
C12—H12	0.9400		
			
N21—Mn1—N21^iii^	176.54 (16)	C11—C12—C13	119.8 (4)
N21—Mn1—N12^i^	91.73 (8)	C11—C12—H12	120.1
N21^iii^—Mn1—N12^i^	91.73 (8)	C13—C12—H12	120.1
N21—Mn1—N11	88.27 (8)	C12—C13—C12^iii^	117.0 (5)
N21^iii^—Mn1—N11	88.27 (8)	C12—C13—C14	121.5 (3)
N12^i^—Mn1—N11	180.0	C12^iii^—C13—C14	121.5 (3)
N21—Mn1—N1	89.88 (12)	N12—C16—C15	123.4 (4)
N21^iii^—Mn1—N1	90.19 (12)	N12—C16—H16	118.3
N12^i^—Mn1—N1	88.79 (8)	C15—C16—H16	118.3
N11—Mn1—N1	91.21 (8)	C16—C15—C14	118.9 (4)
N21—Mn1—N1^iii^	90.19 (12)	C16—C15—H17	120.6
N21^iii^—Mn1—N1^iii^	89.88 (12)	C14—C15—H17	120.6
N12^i^—Mn1—N1^iii^	88.79 (8)	C15^iii^—C14—C15	118.3 (5)
N11—Mn1—N1^iii^	91.21 (8)	C15^iii^—C14—C13	120.8 (3)
N1—Mn1—N1^iii^	177.58 (16)	C15—C14—C13	120.8 (3)
C1—N1—C5	116.9 (3)	C21—N21—Mn1	146.6 (3)
C1—N1—Mn1	120.3 (3)	N21—C21—S21	178.9 (4)
C5—N1—Mn1	122.7 (3)	C32—C31—H31A	109.5
N1—C1—C2	123.8 (4)	C32—C31—H31B	109.5
N1—C1—H1	118.1	H31A—C31—H31B	109.5
C2—C1—H1	118.1	C32—C31—H31C	109.5
C1—C2—C3	119.4 (4)	H31A—C31—H31C	109.5
C1—C2—H2	120.3	H31B—C31—H31C	109.5
C3—C2—H2	120.3	O31—C32—C31	109.5 (10)
C4—C3—C2	117.5 (3)	O31—C32—H32A	109.8
C4—C3—C3^ii^	120.5 (4)	C31—C32—H32A	109.8
C2—C3—C3^ii^	122.0 (5)	O31—C32—H32B	109.8
C3—C4—C5	118.4 (4)	C31—C32—H32B	109.8
C3—C4—H4	120.8	H32A—C32—H32B	108.2
C5—C4—H4	120.8	C32—O31—C33	115.3 (10)
N1—C5—C4	124.0 (4)	O31—C33—C34	110.1 (10)
N1—C5—H5	118.0	O31—C33—H33A	109.6
C4—C5—H5	118.0	C34—C33—H33A	109.6
C11—N11—C11^iii^	116.8 (5)	O31—C33—H33B	109.6
C11—N11—Mn1	121.6 (2)	C34—C33—H33B	109.6
C11^iii^—N11—Mn1	121.6 (2)	H33A—C33—H33B	108.2
C16—N12—C16^iii^	117.1 (5)	C33—C34—H34A	109.5
C16—N12—Mn1^iv^	121.4 (2)	C33—C34—H34B	109.5
C16^iii^—N12—Mn1^iv^	121.4 (2)	H34A—C34—H34B	109.5
N11—C11—C12	123.3 (4)	C33—C34—H34C	109.5
N11—C11—H11	118.4	H34A—C34—H34C	109.5
C12—C11—H11	118.4	H34B—C34—H34C	109.5

Symmetry codes: (i) *x*, *y*−1, *z*; (ii) −*x*, *y*, −*z*+3/2; (iii) −*x*+1, *y*, −*z*+3/2; (iv) *x*, *y*+1, *z*.
